# Pharmacokinetic profile of novel multi-layer stable effervescent tablet: a cross-over study with an established European brand in healthy young male adults

**DOI:** 10.1186/s40360-024-00808-9

**Published:** 2024-11-05

**Authors:** Danish Hassan Dani, Syed Baqir Shyum Naqvi, Muhammad Akram, Matti Ullah, Sheikh Abdul Khaliq, Muhammad Masoom Akhtar, Orva Abdullah, Syed Faisal Badshah, Mohammed Bourhia, Gamal A. Shazly, Yousef A. Bin Jardan, Srosh Fazil

**Affiliations:** 1 Faculty of Pharmacy, Hamdard University Islamabad Campus, Islamabad, Pakistan; 2 Faculty of Pharmacy, Hamdard University-Main Campus, Karachi, Pakistan; 3 Faculty of Pharmacy and Pharmaceutical Sciences, University of Karachi, Karachi, Pakistan; 4 Department of Pharmacy, Faculty of Medical and Health Sciences, University of Poonch, Rawalakot, Pakistan; 5 Swalife Biotech Ltd Unit 3D North Point House, North Point Business Park, Cork, Ireland; 6 Department of Pharmaceutics, College of Pharmacy, King Saud University, Riyadh, 11451 Saudi Arabia; 7 Department of Chemistry, University of Poonch Rawalakot, Rawalakot, Pakistan

**Keywords:** Pharmacokinetic profile, Effervescent tablet, Healthy young male adults, Pakistan

## Abstract

Effervescent formulation helps in faster and better absorption of drugs, especially those that are rapidly soluble in water. However, these tablets require special packaging in order to prevent them from absorbing moisture, hence increasing cost. We compared an effervescent tablet prepared using an in-house developed method (multi-layer tablet with acid and base part separated by an inert layer) to a European effervescent tablet (Efferalgan®) in a single-center, randomized cross-over study among twelve healthy volunteers. Blood samples were collected for 8 h and analyzed for paracetamol concentration using HPLC. Our results showed that both the products have similar pharmacokinetic profiles with no significant difference observed for C_last_, T_half_, K_elim_, and MRT (*p*-value > 0.05). Moreover, to assess bioequivalence we did not find any significant difference *(p-value > 0.05)* in AUC (27.12 ± 6.02 vs. 27.29 ± 2.64 µg.h/ml), C_max_ (7.42 ± 1.06 vs. 7.83 ± 1.19 µg/ml) and t_max_ (0.85 ± 0.22 vs. 0.83 ± 0.25 h). The TR ratios for AUC, C_max_, and t_max_ were 0.99, 0.95, and 1.02 respectively, and were all within the specified FDA limits i.e., 0.8–1.25. We found our test tablet to be bioequivalent to that of Efferalgan®.

**Graphical Abstract:**

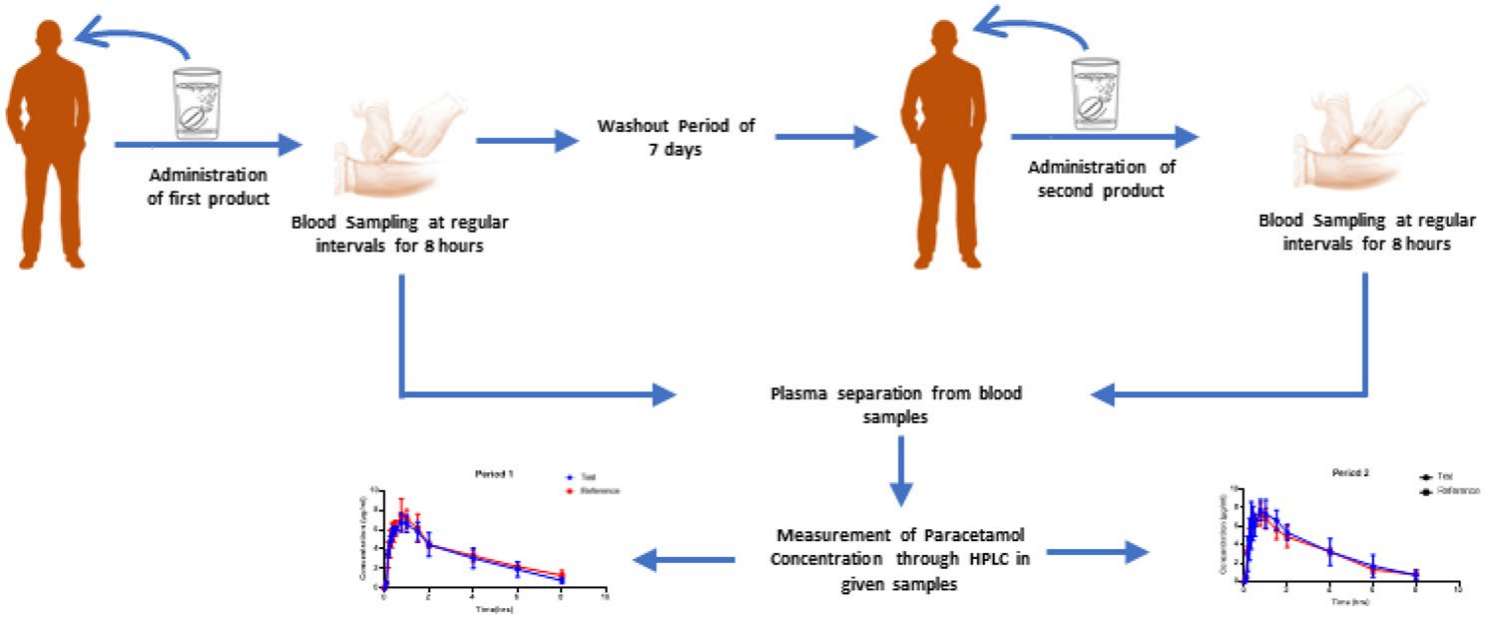

## Introduction

Paracetamol is a widely used analgesic and antipyretic drug that is effective for mild to moderate pain and fever [1]. Compared to other analgesics such as non-steroidal anti-inflammatory medications (NSAIDs) and opioids, it has a good safety profile and a minimal risk of drug interactions [1]. There are many different dosage forms of paracetamol, including tablets, capsules, syrups, suppositories, and injections [2–4]. Effervescent tablets provide the added benefit of easy to administer, and taste masking, and have an early onset of action as compared to conventional tablets. Therefore, these tablets help increase patient compliance [5]. Moreover, although paracetamol is rapidly absorbed from the gastrointestinal tract after oral administration, the first-pass metabolism decreases its availability to the systemic circulation, therefore a rapid absorption of paracetamol is needed [6, 7].

Effervescent tablets do have some drawbacks though, including larger tablets that could be challenging to handle and store, a complicated manufacturing process needing specialized facilities and equipment [8, 9]. The quality and effectiveness of the effervescent tablets might be impacted by environmental factors like humidity, temperature and direct sunlight. As a result, they need to be well protected from these elements using specialized packaging materials and storage settings [10–12].

To address the stability and storage issues, we prepared a formulation that was stable at room temperature for weeks without the need for specialized packaging. Briefly, we prepared a multilayered tablet, to separate acid and base component of tablet to avoid accidental effervescent. Our method exhibited increased shelf-life of the effervescent tablet in accelerated stability studies and the tablets were stable at 75% relative humidity and 40 °C for 6 months [13].

In this study, we compared the pharmacokinetic profile of our in-house developed effervescent (Patent applied; Journal code: 221219 Ref. number: 831/2022) tablet to that of an already marketed European brand i.e., Efferalgan® by UPSA containing 500 mg paracetamol in order to assess bioequivalence of the two products.

## Materials and methodology

### Test tablet preparation and in-vitro characterization

The test tablet was prepared using an in-house developed technique previously published, using method in accordance with ICH GMP guidelines [14]. Briefly, the paracetamol granules were prepared with pre-gelatinized starch and were divided in to two parts. One part was mixed with acid while base was added to other part. The multi-layered tablets were compressed using mannitol as an inert middle layer to keep acid and base components of tablets apart until mixed with water [13]. The tablet was evaluated for physical parameters such as color, shape, hardness, weight variation, effervescent time and assay for paracetamol using UV-spectrometry as discussed by Aslani et al. [11].

### Informed consent and ethical approval

Prior to initiating the study, a written informed consent was obtained from each participant after explaining the research and its objectives. The researchers ensured the maintenance of volunteer data confidentiality in compliance with the 1964 Declaration of Helsinki [15] and followed ICH guidelines [16].

The ethical approval of the study was obtained from the Institutional Bioethics Committee, University of Karachi (Project reference number IBC KU 275/2022). Study is also approved by the Board of Advanced Studies and Research, Hamdard University, Karachi (Reference No. HU/DRA/2018/401).

### Study settings

The study design was open, single-center, randomized, single dose, two-way crossover study involving twelve healthy volunteers administered medicines over two periods with washout time of 7 days [17]. The number of participants and two-way crossover design were selected based on the minimum criteria set by “Guideline on the investigation of bioequivalence” from European Medicines Agency [18]. The test product was in-house developed effervescent tablet and reference product was Efferalgan® (500 mg; UPSA, Lot A2471; Expiry date: November 2024). The patent is already submitted for the product. In period 1, six volunteers (assigned as group A) were given reference product while the other six (assigned as group B) were given test dosage form. In period 2, group A was given the test dosage form while group B used reference product. Blood was sampled from volunteers at 0, 5, 10, 15, 20, 25, 30, 45, 60, 90, 120, 240, 360 and 480 min.

Every individual received the respective product after 12-hours over-night fasting state. Before administering to the patients, the tablets were stirred in water with spoon. In the initial 2 h, volunteers were not allowed to take any food or water. Water was allowed only after 2 h of receiving the product, while a controlled meal of 2 bread slices and a boiled egg was provided to each participant after the 12th blood sampling i.e. after 240 min.

### Sample preparation and analysis

The blood samples were analyzed for paracetamol concentration through HPLC (in-house developed method). The instruments used were HPLC, Nexera, Schimadzu, Japan. Briefly, we used water: Acetonitrile in the ratio of 88:12 as the mobile phase with an injected volume of 10ul and a run time of 7 min. The columns used were 250 × 4.6 mm LC Column C18 with 4 × 3 mm HPLC guard column. The HPLC settings used were a wavelength of 245 nm and flow rate of 1.1 ml/min and retention time of 5 ± 0.5 min. The internal standard used was Acetaminophen (USP standard; USP Catalog no.: 1003009; Lot no.: K2M244). The samples were prepared by taking 4 ml blood samples from each volunteer at specified intervals. Plasma was extracted after getting the vacutainers vortexed for 10 min at 4000 rpm. Plasma was stored in 2 ml Eppendorf tubes after getting them labeled properly. Storage temperature was maintained −20 °C for plasma samples collected throughout the study until the analysis was completed [19].

### Inclusion and exclusion criteria

Male volunteers, having 20–27 years of age and an acceptable weight range within ± 10% of an individual’s ideal body weight, having no previous record of allergies or smoking habits, having normal Blood pressure, LFT, CBC, urine creatinine and other screening criteria. Only those individuals were selected who were vaccinated for COVID 19.

Individuals having any disease, younger than 20 years, abnormal screening tests, obese (BMI > 25), not vaccinated for COVID 19, alcohol or smoking history, blood donated in last 4 months or any administered medicine were excluded from the study.

### Data analysis

Pharmacokinetic parameters were calculated using PK-Solver [20]. Data of Crossover study was analyzed by SPSS-22 (Statistical Package of Social Sciences). Significance was determined in between mean AUC (Area under the plasma-concentration time curve), mean t_max_ (time to achieve maximum concentration) and mean C_max_ (maximum plasma concentration) of test product and reference product by Mann-whitney *t*-test. *P*-value < 0.05 was considered significant. The values are represented as Mean ± S.D.

## Results

The tablets prepared were round in shape and off-white to orange in color. The weight variation, hardness, effervescent time, carbon dioxide content, and solution pH was well within the limits as presented in Table 1. Moreover, the paracetamol assay showed that paracetamol content was at 100.07% and followed the specifications.

**Table 1:** In-vitro characterization of test product

Tests	Specifications	Results
Appearance	Off-white to Orange, round tablet	Comply
Identification (Paracetamol)	Positive	Comply
Assay	90–110%	100.07
Weight variation	1805 mg– 1995 mg	1909.40 mg
Hardness	NLT 5 Kg/cm^2^	6.02 Kg/cm^2^
Effervescent time	< 300 s	120.33 s
Carbon dioxide content	Report results	280.67 mg
Solution pH (After effervescing in 200 ml purified water)	5.0–6.0	5.50

### Comparison of pharmacokinetic profile

The plasma concentration for paracetamol was measured over time in volunteers divided into two groups. The reference group took Efferalgan® and test group taking an in-house prepared formulation. In cross-over study, after washout period, the volunteers were shifted to alternate product.

**Fig. 1:**
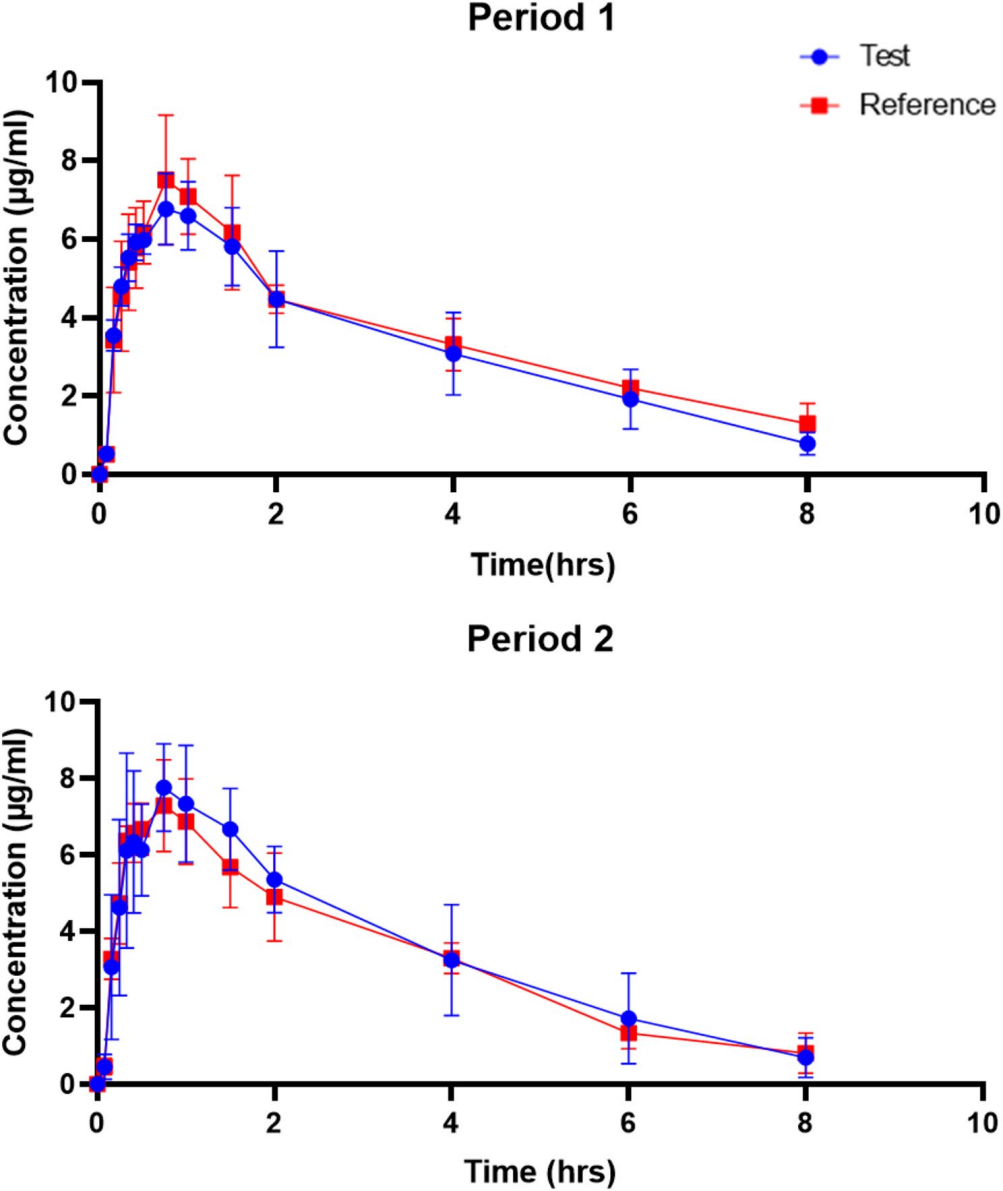
Comparison of plasma concentrations between test and reference group. Period 1 (*top*) and Period 2 (*bottom*). No difference was observed for time required to obtain peak concentration in plasma or overall AUC among both periods

1st Period:

The plasma concentration of the paracetamol in test and reference group over the period of 8 h is shown in Fig. 1 (top). The plasma levels peaked at 45 min for test (6.77 ± 0.91 µg/ml) as well as reference (7.51 ± 1.65 µg/ml) and then started to fall gradually. At the end of 8 h, the mean plasma levels were 1.3 ± 0.52 µg/ml for reference group as compared to 0.79 ± 0.29 µg/ml for test group.

2nd Period:

Figure 1 (bottom) shows the plasma concentration in twelve volunteers for the second period where the volunteers were given alternate product as compared to the product given in first period. Similar, to first period the plasma concentrations peaked at 45 min with 7.77 ± 1.14 µg/ml in plasma of volunteers taking test tablet while 7.29 ± 1.2 µg/ml in plasma of volunteers taking reference product. At the end of 8 h, the plasma concentrations were 0.7 ± 0.52 µg/ml for test group while 0.82 ± 0.53 µg/ml for reference group.

### C_last_, K_elim_, t_half_ and MRT

**Fig. 2:**
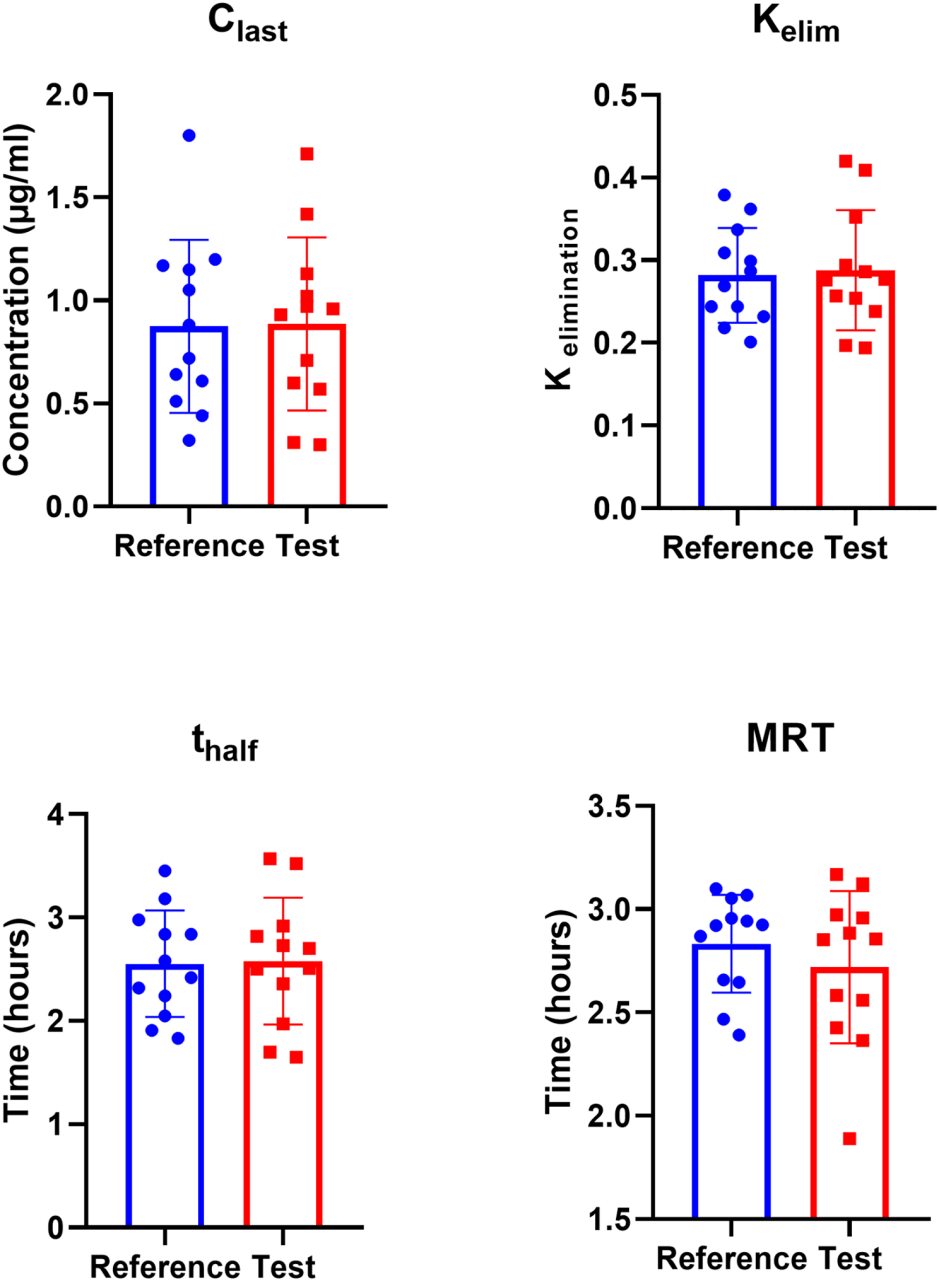
Comparison of pharmacokinetic parameters i.e. C_last_, K_elim_, T_half_ and MRT of test vs. reference. Our test product showed similar PK profile with no difference in any of the studied parameters

No statistical difference was observed between the PK parameters among the two groups. The C_last_, K_elim_, T_half_ and MRT for reference group against test were 0.87 ± 0.42 ug/ml vs. 0.89 ± 0.42 ug/ml, 0.28 ± 0.06 vs. 0.29 ± 0.07, 2.55 ± 0.51 h vs. 2.58 ± 0.61 h and 2.83 ± 0.24 h vs. 2.72 ± 0.37 h, respectively. Therefore, our test group showed similar plasma concentration at the end of the sampling i.e. 8 h with similar residence time, half-life and elimination rate constant, suggesting similar pharmacokinetic profile for our formulation to that of commercially available formulation (Fig. 2).

### Comparison of AUC, C_max_, and t_max_ of test and reference products

**Fig. 3:**
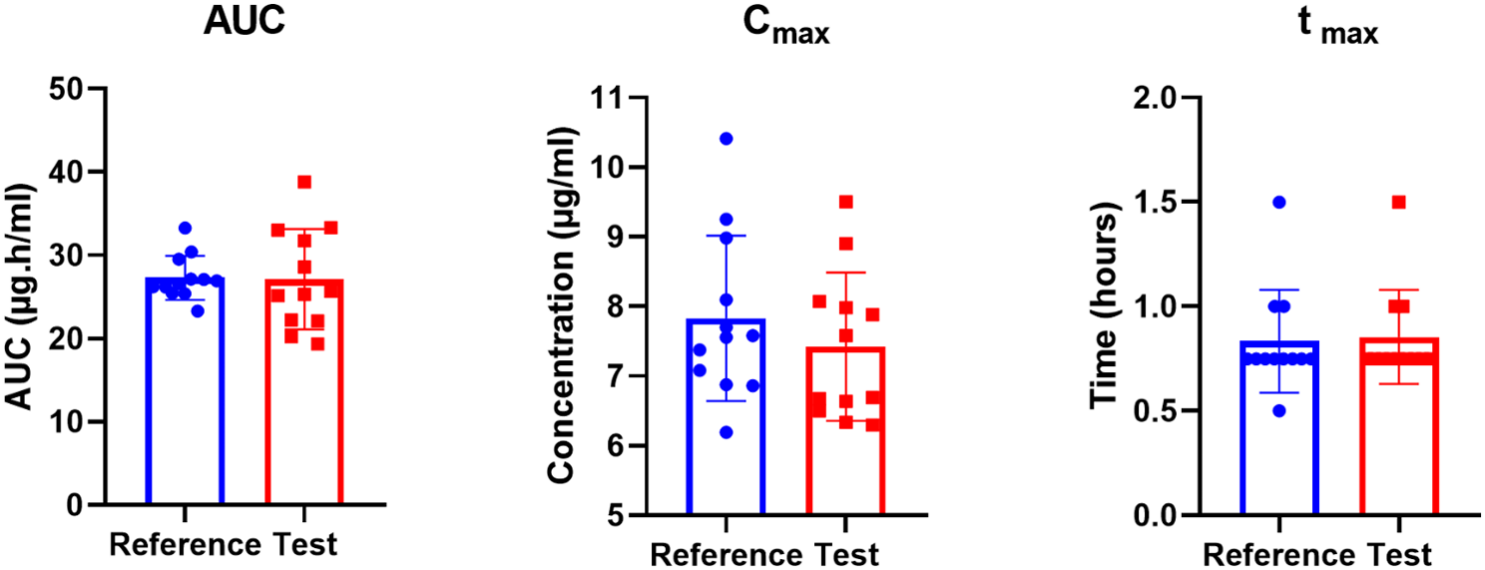
Comparison of PK parameters (AUC, C_max_, t_max_) of test against reference for bioequivalence. No difference was observed for AUC, C_max_ or t_max_ of test product as compared to the reference product

FDA recommends evaluating the AUC, C_max_ and t_max_ of the two products to label them bioequivalent. The calculated values are shown in Table 2; Fig. 3. The AUC for the test group was calculated to 27.12 ± 6.02 and was similar to reference group which was 27.29 ± 2.64 with TR ratio of 0.99. The C_max_ for test group and reference group were 7.42 ± 1.06 vs. 7.83 ± 1.19 with TR ratio of 0.95. The t_max_ was also similar between test and reference group i.e. 0.85 ± 0.22 vs. 0.93 ± 0.25 with TR ratio of 1.02. the p-values for comparison between test and reference group for AUC, C_max_ and t_max_ were 0.93, 0.38 and 0.83, respectively, suggesting similarity between the two formulations. Moreover, all the TR ratios were in accepted criteria i.e., 0.80–1.25.

**Table 2:** Shows a statistical comparison of test and reference products for the area under the curve till time *t*, maximum plasma concentration achieved, and time to reach maximum plasma concentration

Variables	Drug	*N*	Mean ± Std. deviation	Std. error mean	Significance*	TR ratio**
AUC (µg.h/ml)	Test	12	27.12 ± 6.02	1.74	*t* = 0.089, *p* = 0.93	0.99
	Reference	12	27.29 ± 2.64	0.76		
C_max_ (µg/ml)	Test	12	7.42 ± 1.06	0.31	*t* = 0.89, *p* = 0.38	0.95
	Reference	12	7.83 ± 1.19	0.34		
T_max_ (hr)	Test	12	0.85 ± 0.22	0.06	*t* = 0.22, *p* = 0.83	1.02
	Reference	12	0.83 ± 0.25	0.07		

*p-value is considered significant at < 0.05; ** TR = Test to Reference (Recommended Range 0.80–1.25)

## Discussion

Paracetamol can be made into effervescent tablets for better flavor, quicker absorption, and simpler administration [21–23]. Effervescent tablets might lose their quality and effectiveness due to instability and degradation brought on by moisture and carbon dioxide absorption [24, 25]. Previously, we had created a novel method for producing a multi-layer stable effervescent paracetamol tablet with three layers [13]. This design was made with the intention of separating the basic and acidic parts of the effervescent system and shielding them from carbon dioxide and moisture paracetamol [13]. We predicted that using this method would produce an effervescent tablet that was stable and had comparable pharmacokinetics to the reference product. Before administration to volunteers, test tablets were prepared following GMP regulations and it was ensured that the validation criteria are met (Table 1). In a crossover study with twelve healthy volunteers, we compared the pharmacokinetic properties of our test product with those of a known European brand (Efferalgan®) and analyzed bioequivalence between the two products. We took blood samples at regular intervals, and we used HPLC to determine the paracetamol plasma concentration. Then, to determine bioequivalence, we evaluated the pharmacokinetic parameters AUC, C_max_, and t_max_ for each substance and compared them using *t*-test.

Our study’s strength comes from its meticulous planning and execution, which adheres to the global standards (FDA, 2003; EMA, 2010). We measured the plasma concentration of paracetamol with excellent accuracy and precision using a validated HPLC method. In addition, we preserved our samples integrity and quality by keeping them at −20 °C until analysis. Furthermore, to reduce confounding variables that could alter the pharmacokinetics of paracetamol, we chose healthy young male adults who had received the COVID-19 vaccination as the study population.

Our findings demonstrated no significant difference in C_last_, K_elim_, MRT, AUC, C_max_, or t_max_ between the test and reference products’ pharmacokinetic profiles (p-value > 0.05). Further, TR ratios were also all within the FDA-specified range of 0.8–1.25 [26], demonstrating bioequivalence between the two medications (Table 2). These results imply that the therapeutic efficacy of our unique multi-layer stable effervescent paracetamol tablet, which we developed, is interchangeable with the well-known European brand (Efferalgan®). Moreover, we found that the pharmacokinetic parameters of Efferalgan® were in line with the drug profile as reported by the UPSA label, indicating that the analytical techniques used in this study were reliable. Moreover, we did not observe any adverse effect in the volunteers observed over the time period of the study, suggesting that our formulation is safe to be administered to the population. The novel multi-layer stable effervescent tablet may offer potential cost advantages over established brands, making it a more affordable option for healthcare systems in low- and middle-income regions like Pakistan. Its improved stability and ease of use could enhance access to quality medications, benefiting both patients and healthcare providers [27].

The main limitation of our study was that we performed the study in a fasting state and abstained volunteers from taking any medicine 7 days prior to the study. Therefore, our findings do not reflect any drug-food or drug-drug interaction.

## Conclusion

We demonstrated that our in-house developed effervescent tablet has similar pharmacokinetic profile to that of Efferalgan®, an internationally marketed European brand. Therefore, our tablet can be considered as bioequivalent to Efferalgan®.

## Data Availability

All data generated or analyzed during this study are included in this published article.
